# A Gene Encoding Xylanase Inhibitor Is a Candidate Gene for Bruchid (*Callosobruchus* spp.) Resistance in Zombi Pea (*Vigna vexillata* (L.) A. Rich)

**DOI:** 10.3390/plants12203602

**Published:** 2023-10-18

**Authors:** Kitiya Amkul, Kularb Laosatit, Yun Lin, Xingxing Yuan, Xin Chen, Prakit Somta

**Affiliations:** 1 Department of Agronomy, Faculty of Agriculture at Kamphaeng Saen, Kasetsart University, Kamphaeng Saen Campus, Nakhon Pathom 73140, Thailand; fagrkia@ku.ac.th (K.A.); fagrkal@ku.ac.th (K.L.); 2 Institute of Industrial Crops, Jiangsu Academy of Agricultural Sciences, Nanjing 210014, China; linyun881210@163.com (Y.L.); yxx@jaas.ac.cn (X.Y.)

**Keywords:** zombi pea, *Vigna vexillata*, bruchid, seed weevil, *Callosobruchus*, xylanase inhibitor

## Abstract

Two bruchid species, *Callosobruchus maculatus* and *Callosobruchus chinensis*, are the most significant stored insect pests of tropical legume crops. Previously, we identified a major QTL, *qBr6.1*, controlling seed resistance to these bruchids in the cultivated zombi pea (*Vigna vexillata*) accession ‘TVNu 240’. In this study, we have narrowed down the *qBr6.1* region and identified a candidate gene conferring this resistance. Fine mapping using F_2_ and F_2:3_ populations derived from a cross between TVNu 240 and TVNu 1623 (susceptible) revealed the existence of two tightly linked QTLs, designated *qBr6.1-A* and *qBr6.1-B*, within the *qBr6.1*. The QTLs *qBr6.1-A* and *qBr6.1-B* explained 37.46% and 10.63% of bruchid resistance variation, respectively. *qBr6.1-A* was mapped to a 28.24 kb region containing four genes, from which the gene *VvTaXI* encoding a xylanase inhibitor was selected as a candidate gene responsible for the resistance associated with the *qBr6.1-A*. Sequencing and sequence alignment of *VvTaXI* from TVNu 240 and TVNu 1623 revealed a 1-base-pair insertion/deletion and five single-nucleotide polymorphisms (SNPs) in the 5′ UTR and 11 SNPs in the exon. Alignment of the VvTAXI protein sequences showed five amino acid changes between the TVNu 240 and TVNu 1623 sequences. Altogether, these results demonstrated that the *VvTaXI* encoding xylanase inhibitor is the candidate gene conferring bruchid resistance in the zombi pea accession TVNu 240. The gene *VvTaXI* will be useful for the molecular breeding of bruchid resistance in the zombi pea.

## 1. Introduction

The zombi pea (*Vigna vexillata* (L.) A. Rich) is an underutilized legume crop of particular interest that is cultivated in some parts of Africa, India, Australia, and Southeast Asia. The crop is grown principally for its dry edible seeds, but it often also develops edible storage roots that are consumed by inhabitants of these regions [1,2,3,4]. These edible tubers have a high protein content of approximately 15%, which is much higher than the protein contents of potato, yam, and cassava [5]. Bhattacharyya et al. [6] reported that the yield of *V. vexillata*’s tuberous roots can be as high as 7 tons per hectare. Wild forms of *V. vexillata* are widely distributed across tropical and subtropical regions [7]. The zombi pea adapts well to several biotic stresses, including infertile, alkaline, acidic, and saline soils, as well as drought and waterlogging. Further, the zombi pea shows resistance to diseases, insects including bruchids (*Callosobruchus chinensis* L.), *Callosobruchus maculatus* Fab.), and *Zabrotes subfasciatus* Boh.)), bean-pod borers (*Maruca testulalis* Geyer)), pod-sucking bugs (*Clavigralla tomentosicollis* Stal)), and cowpea mottle carmovirus [7].

Bruchids (also known as seed weevils) are stored insect pests that consume the seeds of many species of legumes [8]. Bruchid infestation can cause the total loss of a seed lot within a short period of 2–4 months [9]. Although bruchids are classified as stored insects, they initially infest seeds while legume plants are growing in fields, where female bruchids lay eggs on developing or mature pods; subsequently, the larvae bore through the pods into the seeds to consume the cotyledons and embryo and develop into adults by consuming the nutrients in the seed [9]. When such seeds are harvested and stored, the adult bruchids emerge from them and start new infestations by laying eggs directly on stored seeds [9]. Seeds infested by bruchids cannot be used for human or animal consumption, agricultural use, or trading [9,10,11]. The cowpea weevil (*C. maculatus*) and the azuki bean weevil (*C. chinensis*) are the most significant bruchid species that destroy the seeds of tropical legumes [9]. Although *C. maculatus* originated in Africa and *C. chinensis* originated in Asia, they are now widely distributed in nearly all continents owing to international seed trading. 

The damage caused by the bruchid varies among legume crops, depending on bruchid species and biotypes. *C. maculatus*, for instance, causes yield losses of up to 90% in black gram, 10–78% in pigeon pea, and 4–90% in cowpea [12]. Although several physical and chemical methods can be used to control bruchid infestations, these methods are generally impractical and unsuitable for small landowners and may also increase the cost of production. In addition, chemical methods, including the use of fumigants such as aluminum phosphide, magnesium phosphide, and methyl bromide can lead to chemical contamination and health risks [13]. Consequently, the use of resistant cultivars is the best way to control bruchids. In general, the seeds of most cultivated legume crops are susceptible to bruchids. However, most accessions of cultivated and wild *V. vexillata* are resistant to both *C. maculatus* and *C. chinensis* [14,15]. They also exhibit seed resistance to *Z. subfasciatus*, another bruchid species.

Plant breeders have long been interested in identifying the genes conferring resistance to bruchids in legume crops, so as to understand the mechanism of seed resistance to bruchids in order to efficiently and sustainably breed for resistance. Chotechung et al. [16] finely mapped the *Br* locus controlling resistance to *C. maculatus* and *C. chinensis* in the mungbean (*Vigna radiata* (L.) Wilczek) within a region of 38.0 kb and identified *VrPGIP1* and *VrPGIP2* encoding polygalacturonase inhibitors as candidate genes for the resistance. Similarly, Gamage et al. [17] narrowed down the *qVacBrc2.1* locus conferring resistance to *C. chinensis* in the moth bean (*Vigna aconitifolia* (Jacq.) Maréchal) to a 69.8 kb region and identified *VacPGIP1* and *VacPGIP2* encoding polygalacturonase inhibitors as candidate genes for the resistance. Previously, we identified QTLs controlling resistance to *C. chinensis* and *C. maculatus* in the cultivated zombi pea accession “TVNu 240” [18]. The resistance is expressed in two traits: zero damaged seeds (PDS) and a long developmental period for the adult bruchids (AUDPS), in which three and four QTLs were identified for PDS and AUDPS, respectively, in the *C. chinensis* resistance, while two QTLs were identified for each of the PDS and the AUDPS in the *C. maculatus* resistance. The major QTLs for PDS and AUDPS in *C. chinensis* and *C. maculatus* resistance were in linkage group 6 (LG6) and appeared to be the same locus, designated *qBr6.1* [18]. Nonetheless, causal or candidate genes for resistance have not yet been identified for *qBr6.1*. The identification of causal or candidate genes for a trait is useful for the molecular breeding of crops through marker-assisted breeding and genetic engineering. 

The objectives of this study were to (i) finely locate *qBr6.1* controlling bruchid resistance in TVNu 240 and (ii) identify candidate genes for resistance at *qBr6.1*. The data demonstrated that the locus *qBr6.1* is composed of two closely linked QTLs and that the gene *VvTaXI* encoding a xylanase inhibitor is a candidate gene at *qBr6.1*.

## 2. Results

### 2.1. Variation in Resistance to C. maculatus and C. chinensis in the Parents and F_2_ Population 

**Figure 1:**
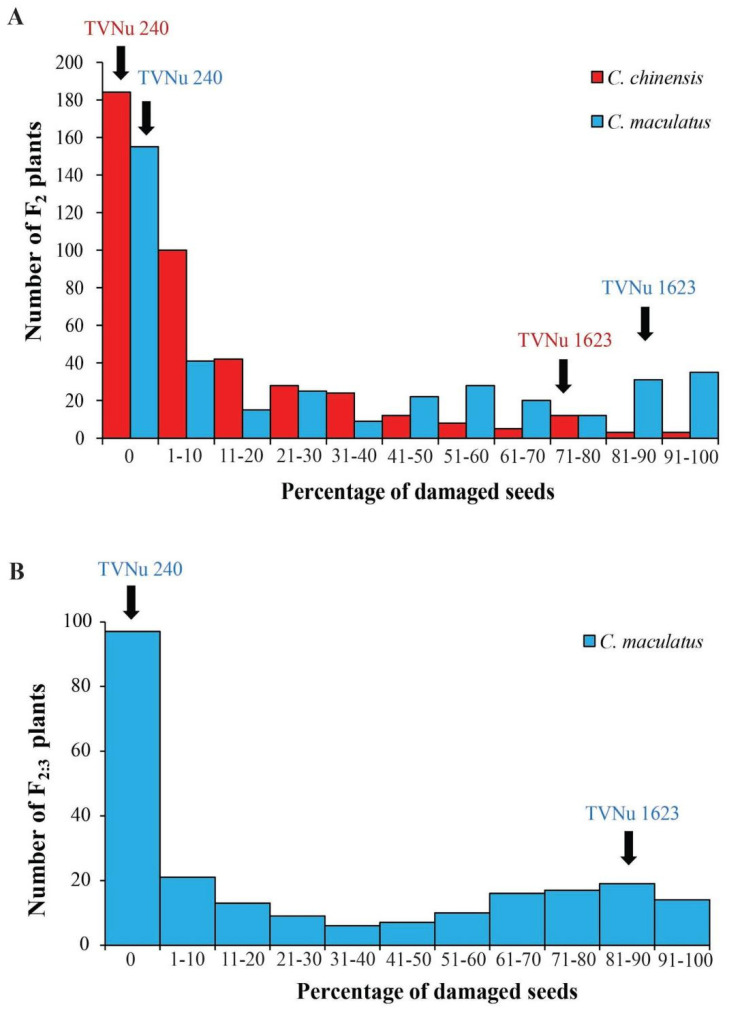
Frequency distribution of the percentage of damaged seeds caused by *C. chinensis* and *C. maculatus* in the F_2_ zombi pea population of a cross between TVNu 240 and TVNu 1623 (**A**) and by *C. maculatus* in the F_2:3_ zombi pea population of a cross between TVNu 240 and TVNu 1623 (**B**).

The F_2_ population and their parents, TVNu 240 and TVNu 1623, were grown in field conditions. The parents showed contrasting responses to both *C. chinensis* and *C. maculatus* infestations. PDS caused by *C. chinensis* in TVNu 240 and TVNu 1623 was 0 and 79.3%, respectively, and 0 and 87.9% in that order by *C. maculatus*. In the F_2_ population the PDS caused by *C. chinensis* varied between 0 and 97.3%, with an average of 13.2%, and that caused by *C. maculatus* ranged from 0 to 100%, with an average of 31.2%. The frequency distributions of PDS caused by *C. chinensis* and *C. maculatus* in the F_2_ population were continuous and skewed towards TVNu 240 (Figure 1A). The correlation between the PDS caused by *C. chinensis* and by *C. maculatus* was significantly high at 0.71 (*p* < 2.2 × 10^−16^).

### 2.2. Fine Mapping qBr6.1 Using the F_2_ Population 

**Figure 2:**
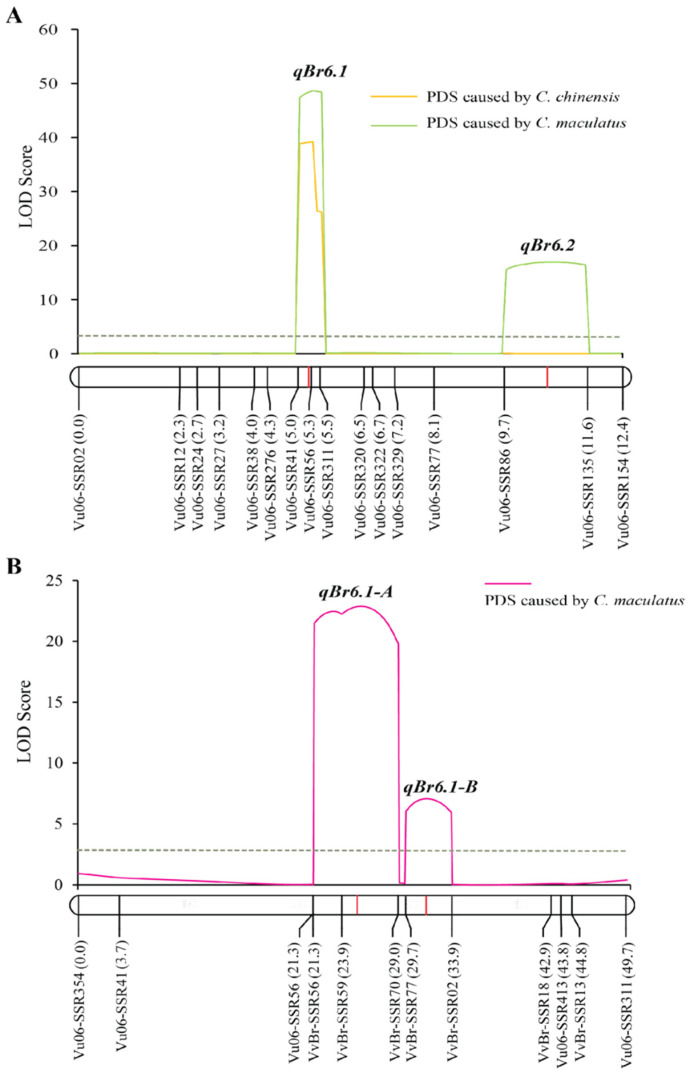
Logarithm of odds (LOD) graph of the QTLs controlling bruchid resistance identified in F_2_ (**A**) and F_2:3_ (**B**) zombi pea populations of a cross between TVNu 240 and TVNu 1623. Dash line horizontal to linkage map is the LOD threshold of the QTLs determined by the permutation test.

Previously, the *qBr6.1* region was mapped between the SNP markers Marker196422 and Marker197124 [18]. Through bioinformatic analysis, these markers were located on the cowpea (*Vigna unguiculata* (L.) Walp.) chromosome 6 at the positions 21,449,068 and 22,216,270, respectively. Thus, these two markers were 767.2 kb apart. A cowpea genome region of 1.0 Mb size covering the *qBr6.1* locus was downloaded for SSR marker development. In total, 311 SSR markers were developed, 33 (10.6%) of which displayed polymorphisms between TVNu 240 and TVNu 1623 (Supplementary Table S1). Sixteen markers showing clear polymorphisms were used to analyze the F_2_ population. A partial linkage map constructed for this population was 12.4 cM in length, with an average distance of 0.83 cM between markers. QTL analysis by the ICIM method showed that only one QTL, *qBr6.1*, controlled the *C. chinensis* resistance, while two QTLs, *qBr6.1* and *qBr6.2*, controlled the *C. maculatus* resistance (Table 1 and Figure 2A). *qBr6.1* was localized between the markers Vu06-SSR41 and Vu06-SSR56. It explained approximately 35% of the total variation in the PDS caused by *C. chinensis* and *C. maculatus* in the F_2_ population. It showed an additive effect of −14.07 and a dominant effect of −10.43 for the PDS caused by *C. chinensis*, and additive and dominant effects of −29.50 and −1.97, respectively, for the PDS caused by *C. maculatus*. The locations of markers Vu06-SSR41 and Vu06-SSR56 were 22,214,142 bp and 22,406,398 bp, respectively; thus, they were 192.26 kb apart in the cowpea genome. 

*qBr6.2* was located between the markers Vu06-SSR86 and Vu06-SSR135. It explained 11.2% of the total variation in the PDS caused by *C. maculatus* in the F_2_ population. This locus exhibited overdominance with an additive effect of −0.25 and a dominant effect of −22.86 for the PDS caused by *C. maculatus*.

### 2.3. Variation in Resistance to C. maculatus in the Parents and F_2:3_ Population 

The F_2:3_ population and its parents, TVNu 240 and TVNu 1623, were grown in field conditions and evaluated for resistance to *C. maculatus*. TVNu 240 and TVNu 1623 exhibited a strong difference in their responses to *C. maculatus* infestation. PDS caused by *C. maculatus* was 0% and 86.2% in TVNu 240 and TVNu 1623, respectively. The PDS in the F_2:3_ population varied from 30.4 to 98.0%, with an average of 30.4%. Similar to the F_2_ population, the frequency distribution of PDS in the F_2:3_ population was continuous and skewed towards the resistant parent, TVNu 240 (Figure 1B). 

### 2.4. Narrowing Down the qBr6.1 Region in the F_2:3_ Population 

To further narrow down *qBr6.1*, SSR markers were developed from the zombi pea reference genome and used to map *qBr6.1* in the F_2:3_ population. A BLASTN search revealed that the markers Vu06-SSR41 and Vu06-SSR56 delimiting *qBr6.1* in the F_2_ population were on the scaffold 0047 (Vigve.0047) of the zombi pea genome at the positions 2,913,204 and 3,187,954, respectively. Thus, these markers were 274.75 kb apart in the zombi pea genome. Nonetheless, a zombie pea genome region of 500.0 kb covering the *qBr6.1* region identified in the F_2_ population was used to develop SSR markers to narrow down *qBr6.1*. One hundred and twenty-one SSR markers were developed and screened for polymorphisms between the mapping parents (Supplementary Table S1). All except 3 markers were amplifiable but only 26 (21.5%) markers showed polymorphisms. Nonetheless, seven zombi pea SSR markers and five cowpea SSR markers were chosen and used to construct the linkage map for the F_2:3_ population. The linkage map was 49.7 cM in length. ICIM revealed that *qBr6.1* was composed of two closely liked QTLs, designated *qBr6.1-A* and *qBr6.1-B*, for the resistance (Figure 2B and Table 2). *qBr6.1-A* was located between markers VvBr-SSR59 and VvBr-SSR70 and explained 37.46% of the total PDS variation caused by *C. maculatus* in the population. It showed an additive effect of −26.59 and a dominant effect of −4.38. On the other hand, *qBr6.1.2* was localized between markers VvBr-SSR77 and VvBr-SSR02. It accounted for 10.63% of the PDS variation in the population and expressed overdominance with additive and dominant effects of −0.95 and −26.11, respectively.

### 2.5. Identification of the Candidate Gene for qBr6.1-A and Sequence Variation in the Candidate Gene 

**Figure 3:**
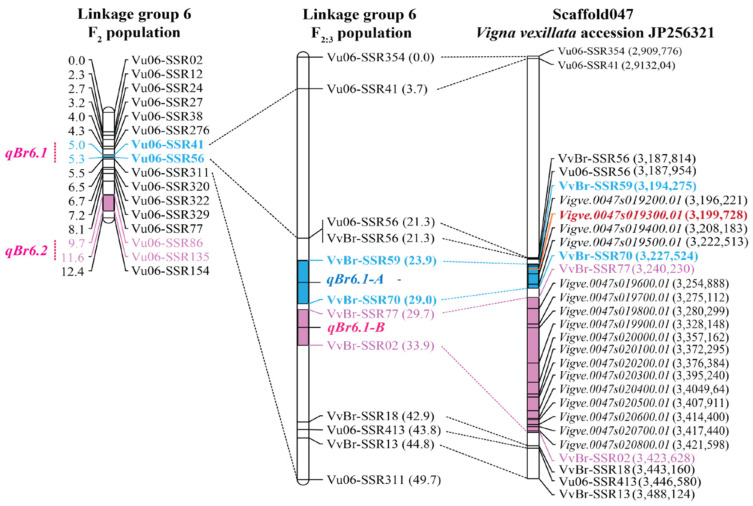
A comparative linkage map illustrating the relationship between QTLs controlling bruchid resistance identified in the F_2_ and F_2:3_ zombi pea population of a cross between TVNu 240 and TVNu 1623 and their physical locations on the zombi pea genome. Only genes existing in the QTL regions *qBr6.1-A* and *qBr6.1-B* are shown. The candidate gene for *qBr6.1-A* is highlighted in red and bolded.

Narrowing down *qBr6.1* in the F_2:3_ population dissected this locus into two closely linked QTLs, *qBr6.1-A* and *qBr6.1-B* (Figure 2B and Table 2). The *qBr6.1-A* region was focused for further study since it showed a much greater genetic effect than *qBr6.1-B*. Gene mapping in the F_2:3_ populations demonstrated that *qBr6.1.1* was delimited to a genome region of 28.24 kb of the scaffold 0047 of the zombi pea reference genome (accession JP256321) (Figure 3). Four genes including *Vigve.0047s019200.01*, *Vigve.0047s019300.01, Vigve.0047s019400.01* and *Vigve.0047s019500.01* were located in this 28.24 kb region (Figure 3 and Table 3). *Vigve.0047s019200.01* and *Vigve.0047s019500.01* were each predicted to encode an uncharacterized protein. *Vigve.0047s019400.01* was predicted to produce a hypothetical protein. *Vigve.0047s019300.01* was annotated to encode a protein containing *Triticum aestivum* xylanase inhibitor (TAXI) domains. This gene was chosen as a candidate gene for bruchid resistance and designated “*VvTaXI*”. 

**Figure 4:**
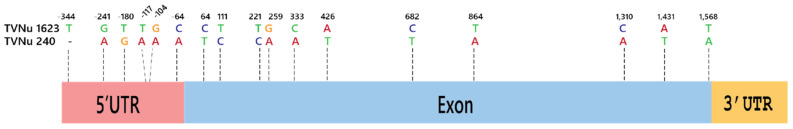
DNA sequence variations in the VvTaXI gene between TVNu 240 and TVNu 1623.

**Figure 5:**
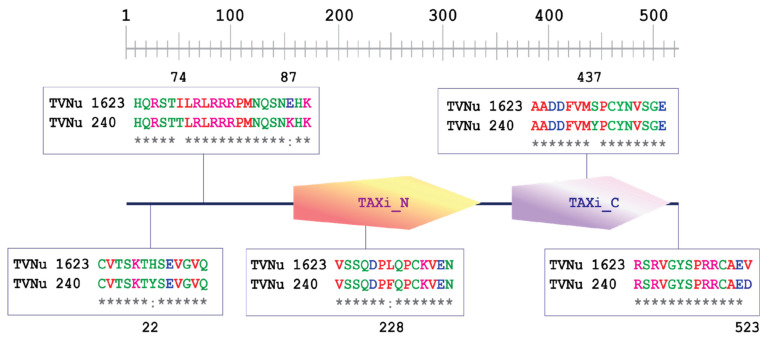
Amino acid sequence variations in VvTaXI protein between TVNu 240 and TVNu 1623. Asterisk indicates conserved amino acid.

According to the zombi pea reference genome, *VvTaXI* is 2177 bp in length and intronless. The *VvTaXI* was amplified from TVNu 240 and TVnu 1623 and sequenced. Sequence alignment revealed a 1-bp insertion/deletion (InDel) and 16 SNPs between *VvTaXI* sequences of TVNu 240 and TVNu 1623 (Figure 4). The InDel and five SNPs were located in the 5′ UTR, while the rest of the SNPs were in the exon. The alignment of the predicted VvTaXI protein sequences in TVNu 240 and TVNu 1623 demonstrated that the length of VvTaXI protein in TVNu 240 and JP256321 was the same (523 amino acid residues) (Figure 5). However, compared to the wild zombi pea TVNu 1623, there were six amino acid changes in TVNu 240 accessions, being at the residues 22 (H → Y), 74 (I → T), 87 (E → K), 228 (L → F), 437 (S → Y), and 523 (V → D). These amino acid changes were caused by the SNP at the positions 64 (C → T), 221 (T → C), 259 (G → A), 682 (C → T), 1310 (C → A), and 1568 (T → A), respectively. The amino acid residues 228 and 437 were in the N- and C-terminal domains of the VvTaXI, respectively (Figure 5). 

In addition to the QTL *qBr6.1-A*, the QTL *qBr6.1-B* was found to contribute to bruchid resistance in TVNu 240 in the F_2:3_ population (Figure 2 and Table 2). *qBr6.1-B* was mapped between the markers VvBr-SSR77 and VvBr-SSR02 corresponding to a region of 183.40 kb of the zombi pea scaffold 0047 (Figure 3). This region harbored 14 genes (Table 3). However, none of the genes is known to be involved in the bruchid or insect resistance.

### 2.6. Expression Analysis of the Gene VvTaXI 

**Figure 6:**
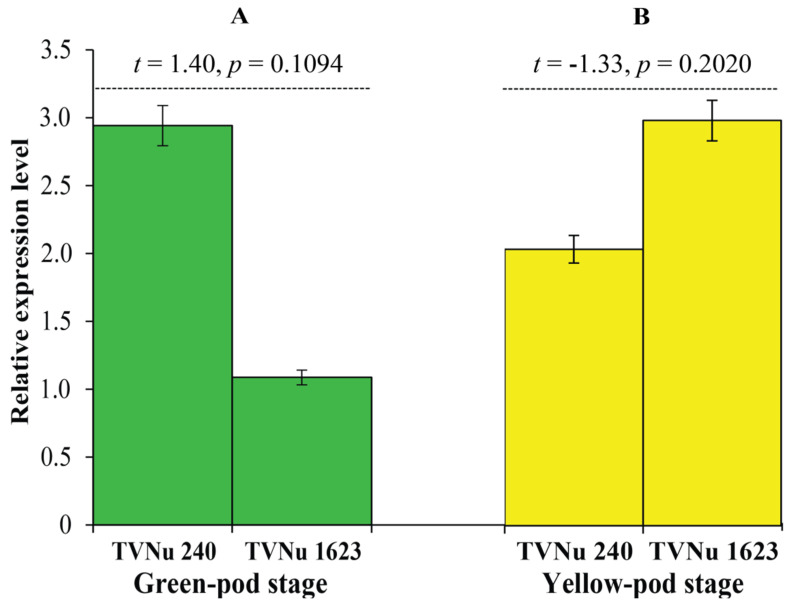
Expression of the *VvTaXI* gene in seeds of TVNu 240 and TVNu 1623 at the green-pod (**A**) and yellow-pod (**B**) stages. The expression is determined by RT-qPCR. Error bar is standard error.

RT-qPCR analysis was conducted to measure the expression level of *VvTaXI* in the seeds of TVNu 240 and TVnu 1623 at two stages, green and yellow pods. RT-qPCR analysis revealed that there was no statistically significant difference in *VvTaXI* gene expression between the TVNu 240 and TVnu 1623 at both stages (Figure 6).

## 3. Discussion

Zombi pea has long received attention from plant breeders and entomologists as a source of bruchid resistance owing to the prevalence of high resistance in both cultivated and wild forms of zombi pea against bruchids. However, the underlying mechanism of the resistance has been poorly understood. To elucidate this mechanism, Birch et al. [14] suggested that p-aminophenylalanine (PAPA) in seeds of zombi pea is a principal defense chemical against *C. maculatus* and *Z. subfasciatus*, which was countered by Bressan [15]. Lattanzio et al. [19] suggested that a high level of α-amylase inhibitor may be associated with the resistance in zombi pea. In our previous study, based on comparative genome analysis using common bean (*Phaseolus vulgaris* L.), we showed that the major locus *qBr6.1* conferring the resistance was distantly linked (approximately 750 kb apart) with genes producing α-amylase inhibitors, suggesting that the α-amylase inhibitor is very unlikely to be the cause of the bruchid resistance. However, in this study, we exploited the cowpea reference genome [20] (https://phytozome-next.jgi.doe.gov/info/Vunguiculata_v1_2 (accessed on 21 January 2020)) and the recently available zombi pea reference genome [21] (https://viggs.dna.affrc.go.jp (accessed on 25 May 2021)) to narrow down the *qBr6.1* region. Zombi pea and cowpea are genetically closely related species [22], although they belong to different subgenera of the genus *Vigna* [23]. A previous comparative study demonstrated that the genomes of these two species are highly conserved [18]. The results of the present study showed that the *qBr6.1* locus consists of two tightly linked QTLs, *qBr6.1-A* and *qBr6.1-B* (Figure 2B and Table 2), and that the gene *VvTaXI* encoding a TAXI-type xylanase inhibitor may be responsible for bruchid resistance at *qBr6.1-A* (Figure 3, Figure 4 and Figure 5).

Bruchids are phytophagous insects that feed exclusively on cotyledons of legume seeds [8,24]. Seed cotyledons have a cell wall composed mainly of a heterogeneous mixture of hemicellulose polysaccharides, including arabinoxylans. Coleopteran insects including seed bruchids/weevils that feed on seeds or cotyledons utilize plant cell wall-degrading enzymes (PCWDEs), for example polygalaturonases, amylases and xylanases, to depolymerize the structural polysaccharides in order to invade, attack and digest plant tissues for their nutrients [25,26]. To defend themselves from bruchids, plants produce and utilize several enzyme inhibitors against these PCWDEs. Protein inhibitors of PCWDEs and starch such as polygalacturonase inhibitors and α-amylase inhibitors have been shown to provide resistance to *C. chinensis* and *C. maculatus* [27,28]. Xylans are the most common hemicelluloses and considered to be the second most abundant polysaccharides in plants. They account for up to 50% of the weight of seed tissues of some plant species [29]. Xylanase is an enzyme that is involved in the depolymerization of xylan into simple monosaccharides and xylooligosaccharides [30]. An enzyme present in the midgut of *C. maculatus* has been reported to exhibit exclusive xylanase activities [25]. Three types of xylanase inhibitors have been identified and characterized, viz., TAXI, xylanase inhibitor protein (XIP), and thaumatin-like xylanase inhibitors (TLXI) (for review, see [31]). The involvement of these xylanase inhibitors in plant defense against insects and pathogens has been established by several reports (for review, see [31,32]).

TAXIs are xylanase inhibitors that inhibit family 11 endo-β-1,4-xylanase, which is a PCWDE produced and used by phytophagous insects, including bruchids, to utilize plants as sources of nutrients for their growth and development. Recently, Yan et al. [33] reported that the *PsXI* gene encoding a TAXI-type xylanase inhibitor is the candidate to confer resistance against *C. chinensis* and *C. maculatus* in pea (*Pisum sativum* L.) seeds. They showed that pea with the *PsXI* gene encoding a complete xylanase inhibitor are resistant to those bruchids, whereas pea with a form of *PsXI* gene encoding a truncated xylanase inhibitor are susceptible to them. Similar results were found in our study, where the bruchid-resistant zombi pea TVNu 240 and the bruchid-susceptible zombi pea TVNu 1623 possessed different *VvTaXI* alleles (Figure 4) that produce VvTaXI proteins with different amino acid sequences (Figure 5). This difference in amino acid sequences between TVNu 240 and TVNu 1623 was within the TAXi_N and TAXi_C domains (Figure 5), both of which are necessary for creating the catalytic pocket for cleaving xylanases [34,35,36,37]. This suggests that, as compared to TVNu 1623, the amino acid changes in the TAXi_N and TAXi_C domains in TVNu 240 result in functional *VvTaXI* with the ability to degrade/inhibit the xylanase activity of the bruchids, and thus seed resistance to bruchids. 

## 4. Materials and Methods

### 4.1. Plant Materials

Two mapping populations, F_2_ and F_2:3_, were used in this study. Both populations were developed from the cross between TVNu 240 and TVNu 1623. TVNu 240 is a cultivated zombi pea that is resistant to *C. maculatus* and *C. chinensis*, while TVNu 1623 is a wild zombi pea susceptible to both *C. maculatus* and *C. chinensis*. F_2_ was comprised of 427 plants. It is worth noting that the population used in the QTL analysis of bruchid resistance in Amkul et al. [18] was a subset of the F_2_ population used in this study. The F_2_ plants and 10 plants of each of their parents were grown under field conditions at Kamphaeng Saen, Nakhon Pathom, Thailand, from December 2017 to March 2018. The spacing between the plants was 0.75 × 0.75 m. Mature pods/seeds from each plant were harvested and used for bruchid resistance evaluation. The F_2:3_ population comprised 218 F_3_ plants derived from 16 F_2_ plants showing a heterozygous genotype at the SNP markers Marker106138 and Marker106117 flanking the QTL *qBr6.1* [18]. A total of 12 to 28 F_3_ plants from each F_2_ plant were combined into the F_2:3_ population. The population and their parents were grown under the same field conditions used to grow the F_2_ population, using the same spacing, from December 2020 to March 2021. Mature pods/seeds from each plant were harvested and evaluated for bruchid resistance.

Genomic DNA of the F_2_ and F_2:3_ plants was extracted using the CTAB method as per the procedures described by Lodhi et al. [38]. The quality and quantity of the DNA were measured through 1.0% agarose gel electrophoresis by comparing with a known concentration of lambda DNA.

### 4.2. Evaluation of Seed Resistance to Bruchids

Evaluations of seed resistance to bruchids were conducted at the Institute of Industrial Crops, Jiangsu Academy of Agricultural Sciences, Nanjing, China. In the F_2_ population, the evaluation was performed using both *C. chinensis* and *C. maculatus* following the same procedures described by Amkul et al. [18]. The bruchids were reared in mass in large plastic containers using mungbean seeds. Fifty mature and intact seeds of each plant were put into a small plastic box. Twenty-five pairs of adults bruchids (25 males and 25 females) that emerged from the mungbean seeds were introduced into the box. The adult bruchids were maintained in the box for egg laying for seven days at 28 °C and 60% relative humidity. Fifty days after insect introduction, the number of seeds damaged by the bruchids (seeds with holes) was counted and the percentage of damaged seeds (PDS) was calculated.

In the F_2:3_ population, the resistance evaluation was carried out using only *C. maculatus* following the same procedure used for the F_2_ population.

### 4.3. DNA Marker Development and Analysis, and QTL Analysis in the F_2_ Population

Initially, as the reference genome sequence of zombi pea was not available, the reference genome sequence of cowpea (*Vigna unguiculata*) [20], a closely related species of zombi pea, was used to develop simple sequence repeat (SSR) markers. The DNA sequence of the SNP markers Marker197124 and Marker196422 flanking *qBr6.1* controlling the resistance to *C. chinensis* and *C. maculatus* [18] was subjected to BLASTN [39] against the cowpea reference genome v.2.1 (https://phytozome-next.jgi.doe.gov/info/Vunguiculata_v1_2 (accessed on 21 January 2020)). A cowpea genome sequence of 1.0 Mb covering the Marker197124 and Marker196422 was downloaded and identified for SSRs using the software SSRIT [40]. Primers for the SSRs were designed using the software Primer3 [41]. The primers were screened for polymorphisms between the mapping parents, TVNu 240 and TVNu 1623. SSR marker analysis (polymerase chain reaction (PCR), gel electrophoresis, and visualization of DNA bands) was carried out as per Laosatit et al. [42]. Markers showing clear polymorphisms were used to analyze the F_2_ population.

The linkage map of the F_2_ population was constructed using QTL IciMapping 4.1 software [43]. Markers were grouped with a logarithm of the odds (LOD) value of 3.0 and ordered using the REcombination Counting and ORDering (RECORD) algorithm [44]. The QTL controlling bruchid resistance was located onto the linkage map using the inclusive composite interval mapping (ICIM) method [45] available in the software QTL IciMapping 4.1. ICIM was conducted at every 0.1 cM with a probability in stepwise regression (PIN) of 0.001. A 3000-permutation test at a probability level of 0.01 was performed in order to determine the optimum LOD threshold for the QTLs.

### 4.4. DNA Marker Development and Analysis, and QTL Analysis in the F_2:3_ Population

After the *qBr6.1* was narrowed down in the F_2_ population, the reference genome sequence of zombi pea became available [21] (http://viggs.dna.affrc.go.jp (accessed on 25 May 2021)). Therefore, the *qBr6.1* was further narrowed down using the F_2:3_ population. We performed a BLASTN search of the sequences of SSR markers located around the *qBr6.1* detected in the F_2_ population against the zombi pea reference genome. A 500.0 kb region of the zombi pea genome was downloaded and used to develop SSR markers as described above. The marker polymorphism screening and analysis were the same as that in the F_2_ population. All polymorphic SSR markers developed from zombi pea and some polymorphic SSR markers developed from cowpea were used to analyze the F_2:3_ population. Subsequently, linkage map construction and QTL analysis of the F_2:3_ population were performed following the same procedures described for the F_2_ population.

### 4.5. Identification and Sequencing of a Candidate Gene Controlling Bruchid Resistance

After the QTL for bruchid resistance was finely mapped in the F_2:3_ population, a candidate gene controlling the resistance was identified by determining the location of DNA markers associated with the resistance QTL on the zombi pea reference genome sequence [21] (https://viggs.dna.affrc.go.jp (accessed on 25 May 2021)) through BLASTN [35]. Subsequently, annotated gene(s) residing in the QTL region with functions that may be associated with seed resistance to bruchids were chosen as candidate gene(s). The gene *Vigve.0047s019300.01* in TVNu 1623 and TVNu 240 was amplified and sequenced using primers listed in Supplementary Table S1. PCR was carried out in a total volume of 10 μL containing 5 ng of DNA template, 1 × *Taq* buffer, 2 mM MgCl_2_, 0.2 mM dNTPs, 1 U KOD-Plus-Neo DNA polymerase (TOYOBO, Beijing, China), and 0.5 μM each of the forward and reward primers. PCR was performed in a SimpliAmp thermal cycler (Applied Biosystems, Waltham, MA, USA) programmed at 94 °C for 2 min followed by 35 cycles of 94 °C for 30 s, 55 °C for 30 s, 72 °C for 1 min, and 72 °C for 10 min. PCR products were checked on 1.5% agarose gel electrophoresis and were Sanger sequenced using the ABI 3730xl DNA Analyzer (Applied Biosystems, United States) by 1st BASE (Singapore).

The sequences of TVNu 1623 and TVNu 240 were aligned to identify nucleotide polymorphism(s) using Clustal Omega [46]. In addition, the coding sequence (CDS) of *Vigve.0047s019300.01* in TVNu 240 and TVNu 1623 were translated into protein sequences and aligned to find amino acid polymorphism(s).

### 4.6. Gene Expression Analysis of the Candidate Gene 

TVNu 240 and TVNu 1623 were grown in a crossing block. Total RNA was extracted from the seeds collected at the green- and yellow-pod stages of these two accessions using the method described by Laksana and Chanprame [47]. cDNA synthesis and quantitative real-time PCR (RT-qPCR) were performed as per Laosatit et al. [38] with an exception that the RT-qPCR was performed using the ViiA 7 Real-Time PCR System (Applied Biosystems, United States). Primers used for the RT-qPCR are listed in Supplementary Table S1. The expression level of the *Vigve.0047s019300.01* was calculated based on the ΔC_T_ method by using *ACTIN* as the reference [48]. A significant difference in the gene expression was tested by Student’s t-test at a probability level of 0.01.

## Figures and Tables

**Table 1:** Location and effects of the QTLs *qBr6.1* and *qBr6.2* controlling resistance to *C. chinensis* and *C. maculatus*. The QTLs are identified in the F_2_ population of the cross TVNu 240 × TVNu 1623 by the inclusive composite interval mapping method.

Bruchid Species	QTL Name	Position on Linkage Group 6 (cM)	Marker Interval	LOD Score	PVE (%)	Additive Effect	Dominant Effect
*C. chinensis*	*qBr6.1*	5.3	Vu06-SSR41–Vu06-SSR56	39.20	34.78	−14.07	−10.43
*C. maculatus*	*qBr6.1*	5.3	Vu06-SSR41–Vu06-SSR56	48.66	34.94	−29.50	−1.97
	*qBr6.2*	11.2	Vu06-SSR86–Vu06-SSR135	16.93	10.42	−0.25	−22.86

PVE = the percentage of variance explained by the QTL.

**Table 2:** Location of effects of the QTLs *qBr6.1-A* and *qBr6.1-B* controlling resistance to *C. maculatus*. The QTLs are identified in the F_2:3_ population of the cross TVNu 240 × TVNu 1263 by inclusive composite interval mapping method.

QTL Name	Position on Linkage Group 6 (cM)	Marker Interval	LOD Score	PVE (%)	Additive Effect	Dominant Effect
*qBr6.1-A*	25.6	VvBr-SSR59–VvBr-SSR70	22.89	37.46	−26.59	−4.38
*qBr6.1-B*	31.6	VvBr-SSR77–VvBr-SSR02	7.07	10.63	−0.95	−26.11

**Table 3:** Annotated genes in *qBr6.1-A* and *qBr6.1-B* regions controlling bruchid resistance in zombi pea (*V. vexillata*) accession TVNu 240.

QTL	Gene	Scaffold	Location	Description
*qBr6.1-A*	*Vigve.0047s019200.01*	0047	3196221..3196999 (+strand)	Uncharacterized protein
	*Vigve.0047s019300.01*	0047	3199728..3201904 (−strand)	TAXI protein
	*Vigve.0047s019400.01*	0047	3208183..3217005 (+strand)	Hypothetical protein
	*Vigve.0047s019500.01*	0047	3222513..3224711 (+strand)	Uncharacterized protein
*qBr6.1-B*	*Vigve.0047s019600.01*	0047	3254888..3256167 (−strand)	Isopenicillin N synthase-like
	*Vigve.0047s019700.01*	0047	3275112..3275345 (−strand)	Hypothetical protein
	*Vigve.0047s019800.01*	0047	3280299..3281578 (−strand)	Isopenicillin N synthase-like
	*Vigve.0047s019900.01*	0047	3328148..3348849 (+strand)	Dentin matrix acidic phosphoprotein 1-like
	*Vigve.0047s020000.01*	0047	3357162..3363147 (−strand)	N-acylphosphatidylethanolamine synthase
	*Vigve.0047s020100.01*	0047	3372295..3376044 (+strand)	Hypothetical protein
	*Vigve.0047s020200.01*	0047	3376384..3376962 (−strand)	Protein PXR1
	*Vigve.0047s020300.01*	0047	3395240..3404186 (+strand)	Chloroplastic protein TIC 40
	*Vigve.0047s020400.01*	0047	3404965..3407557 (+strand)	Uncharacterized protein
	*Vigve.0047s020500.01*	0047	3407911..3411731 (−strand)	WAT1-related protein At3g02690
	*Vigve.0047s020600.01*	0047	3414400..3416005 (+strand)	Late embryogenesis abundant protein
	*Vigve.0047s020700.01*	0047	3417441..3419499 (+strand)	Protein ROOT PRIMORDIUM DEFECTIVE 1
	*Vigve.0047s020800.01*	0047	3421598..3422730 (−strand)	Uncharacterized protein

## Data Availability

Data will be made available on request. The *VvTaXI* sequences generated in this study were deposited to the National Center for Biotechnology Information (GenBank) (https://www.ncbi.nlm.nih.gov (accessed on 20 August 2023)) (accession number OR395213 and OR395214).

## Supplementary Materials

The following supporting information can be downloaded at: https://www.mdpi.com/article/10.3390/plants12203602/s1, Table S1: Primer sequences used in this study and their amplification and polymorphism in zombi pea accessions TVNu 240 and TVNu 1623.
